# Diversity and Abundance of Bacterial and Fungal Communities Inhabiting *Camellia sinensis* Leaf, Rhizospheric Soil, and Gut of *Agriophara rhombata*

**DOI:** 10.3390/microorganisms11092188

**Published:** 2023-08-30

**Authors:** Hao Qu, Yaqin Long, Xuesong Wang, Kaibo Wang, Long Chen, Yunqiu Yang, Linbo Chen

**Affiliations:** 1 Tea Research Institute, Yunnan Academy of Agricultural Sciences, Kunming 650000, China; 2 Yunnan Provincial Key Laboratory of Tea Science, Tea Research Institute, Yunnan Academy of Agricultural Sciences, Menghai 666201, China; 3 State Key Laboratory of Tea Plant Biology and Utilization, Anhui Agricultural University, Hefei 230000, China

**Keywords:** metagenomics, bacterial and fungal diversity, *Agriophara rhombata*, *Camellia sinensis*, gut microbiome

## Abstract

*Agriophara rhombata* is a tea leaf moth that is considered one of the most destructive pests of *Camellia sinensis* (tea plant). Several recent studies have shown that many insects acquire part of the microbiome from their host and soil, but the pattern and diversity of their microbiome have not been clearly demonstrated. The present study aimed to investigate the bacterial and fungal communities present in the rhizospheric soil and leaf of tea plant compared to the gut of tea moth at different developmental stages (larvae, pupae, adult female and male) using Illumina MiSeq technology. Alpha diversity (Shannon index) showed higher (*p* < 0.05) bacterial and fungal diversity in soil samples than in leaf and tea moth larvae, pupae, and adult gut samples. However, during different developmental stages of tea moth, bacterial and fungal diversity did not differ (*p* > 0.05) between larvae, pupae, female, and male guts. Beta diversity also revealed more distinct bacterial and fungal communities in soil and leaf samples compared with tea moth gut samples, which had a more similar microbiome. Furthermore, Proteobacteria, Firmicutes, and Tenericutes were detected as the dominant bacterial phyla, while Ascomycota, Basidiomycota, and Mortierellomycota were the most abundant fungal phyla among all groups, but their relative abundance was comparatively higher (*p* < 0.05) in soil and leaf samples compared to tea moth gut samples. Similarly, *Klebsiella*, *Streptophyta*, and *Enterococcus* were the top three bacterial genera, while *Candida*, *Aureobasidium*, and *Strelitziana* were the top three fungal genera, and their relative abundance varied significantly (*p* < 0.05) among all groups. The KEGG analysis also revealed significantly higher (*p* < 0.5) enrichment of the functional pathways of bacterial communities in soil and leaf samples than in tea moth gut samples. Our study concluded that the bacterial and fungal communities of soil and tea leaves were more diverse and were significantly different from the tea moth gut microbiome at different developmental stages. Our findings contribute to our understanding of the gut microbiota of the tea moth and its potential application in the development of pest management techniques.

## 1. Introduction

*Camellia sinensis* (L.) *Kuntze*, commonly known as the tea plant, belongs to the Theaceae family and is widely cultivated worldwide for its leaves. It is a very important non-alcoholic beverage and is becoming popular as an essential health drink [1]. It grows well in tropical and sub-tropical regions with adequate rainfall, drainage, and mildly acidic soil [2]. There are two main groups of tea plants: *Camellia sinensis* var. *sinensis* (China tea) is predominant in China, Taiwan, and Japan, while *C*. *sinensis* var. *assamica* (Assam tea) is cultivated extensively in Southeast Asia and more recently in Australia [3]. The leaves of the tea plant are processed into a wide range of tea beverages including green, black, dark, white, oolong, and yellow teas with unique flavors and aromas [4]. It offers significant health and medicinal benefits to humans due to its unique secondary metabolites with antioxidant properties, including caffeine, theanine, catechins, theaflavins, thearubigins, flavonoids, and polyphenols [5,6,7,8]. The tea plant is endemic to southwestern China, but it is now cultivated in more than 60 countries as an important green cash crop, covering approximately five million hectares [9,10]. China, India, Kenya, and Sri Lanka are the major tea-producing countries, accounting for 86% of the total tea production, followed by Vietnam, Turkey, Indonesia, Japan, Iran, and Argentina [11,12]. Among them, China alone contributes 3.18 million metric tons, which covers 44% of the global tea production annually [10,12,13]. It has positively influenced the growth of China’s agricultural economy. Unfortunately, tea production is affected by various biotic and abiotic factors, resulting in enormous quality degradation and yield losses worldwide [14,15]. 

Tea plants are susceptible to over 250 insect species that cause 5–55% or sometimes 100% yield loss. Among them, Lepidoptera is the most common group of pests that defoliate tea leaves and cause yield losses of up to 40% [16,17]. In China, 29 Lepidoptera species are a major threat to tea gardens, causing devastating economic losses [18]. *Agriophara rhombata* (Tea ash wood moth), also known as the tea grain moth is one of the most destructive pests that cause leaf damage to tea plants. Once a major pest in India, the tea moth has now been documented in several provinces of China, including Yunnan, Hainan, Fujian, Taiwan, and Guangdong [19]. Tea moth larvae spin silk on the leaves to form buds, hide in these buds, and feed on the mesophyll of the mature plant leaves. They may even consume the bark of the leaves and twigs during the feeding period, which is detrimental to plant vigor and yield [19]. 

Soil and plant microbiomes possess a high diversity of bacteria and fungi, and their microbiomes are linked because the plants are soil-rooted so a part of the soil microbiome inhabits the plant roots [20,21]. Furthermore, the upper parts of plants (leaves and stems) are also inhabited by certain microorganisms, either pathogenic or beneficial [22]. In addition, insects are associated with several microbes. While feeding on the plant host, these insects ingest microbes from the plant and also from the soil via the plant or directly from the roots, which can be assimilated into the insect’s microbiome [23]. The gut microbiota plays a critical role in the growth, development, and ability of an organism to withstand its environment [24]. In many insect species, it facilitates growth, provides additional nutrients, defends against pathogens, and degrades toxins. The gut microbiota reflects the host physiology and varies among different insect groups, gut regions, and life cycle stages [25]. Lepidoptera are a highly diverse group of insects and their gut microbiome exhibits high levels of variability in terms of microbial composition and diversity [26]. Lepidoptera often consume a significant amount of plant material during development, so their guts are filled with a good deal of plant biomass. According to previous research, Lepidopteran species exhibit significant differences in the gut microbiota [27]. The environment, insect nutrition, insect developmental stage, and gut physiology are some of the factors that may operate alone or jointly to produce the great heterogeneity of the lepidopteran gut microbiome [27]. Despite being essential parts of the majority of terrestrial foodwebs and having incredible diversity, the significance of microbiome in Lepidoptera is still unclear [28]. Metagenomics has emerged as a powerful tool for characterizing the diversity of microbial communities inhabiting the insect gut. It allows comprehensive analysis of microbial diversity without culturing individual species [29]. In the present study, 16S rRNA and ITS amplicon sequencing was used to analyze the dynamics of gut microbial communities (bacteria and fungi) at different developmental stages of the tea grain moth, the soil they inhabit, and the relationship between soil and tea microorganisms.

## 2. Materials and Methods

### 2.1. Sampling Site and Data Collection

The study was conducted in Puer, Yunnan, China. The present study compared the microbial communities (bacteria and fungi) present in the gut of *Agriophara rhombata* (larva, pupa, male, and female stages) and the leaves and rhizosphere soil of *Camellia sinensis* plant. The completely randomized design (CRD) with six treatments (larva, pupa, male, female, leaves, and rhizosphere soil) was adopted, with each treatment having five replicates. The detailed treatments and their replicates are listed in Table S1. For the gut microbiome, infested leaves of *C. sinensis* (tea plant) were taken from five random sun-exposed tea plants. Tea plant leave were transported in a cold (8 °C) container and promptly dissected to collect tea moth life stages such as larva, pupa, and male and female adults. Multiple contaminated leaves were dissected to gather 5 individuals for each life stage. All life stages were surface-sterilized in 96% ethanol for 30 s, followed by treatment with 5.2% sodium hypochlorite for 30 s, and rinsed three times in sterile PBS buffer (0.137 M NaCl; 2.7 mM KCl; 10 mM Na_2_HPO_4_; 1.8 mM KH_2_PO_4_; pH 7.4). The intestines from larvae, pupae, and female and male adults were dissected separately under sterile conditions as follows: the anterior end (head) of the adults (male and female) was held by a fine sterile dissecting needle and the posterior end was gently pulled with another sterile dissecting needle until the intact gut was exposed under a drop of sterile PBS buffer. The dissected intestines were stored in PBS at 4 °C until being further processed for DNA isolation. Due to limitations in gut dissection, such as the tiny size of the larva and the pupa, surface-sterilized complete bodies of the larva and pupa were employed separately for total DNA isolation. For the leaf samples, the swab method was used as reported previously [30]. For the soil samples, a five-point sampling method was used. The soil samples were promptly kept in sterile plastic bags, placed in iceboxes, and sent to the laboratory. The samples were then thoroughly homogenized after being processed through a 2-mm sieve.

### 2.2. DNA Extraction, Amplification, and Illumina Sequencing of ITS and 16S rRNA Amplicons

DNA from tissue samples obtained from the larval gut, pupal gut, male and female moth gut, and tea leaves was extracted using the MP Biomedical FastDNA^TM^ Spin Kit following the manufacturer’s protocol. In the case of soil samples, a Qiagen DNeasy PowerSoil Kit (Hilden, Germany) was used for DNA extraction. The extracted DNA was then assessed for its quality using 0.8% agarose gel and was quantified using a Nanodrop-1000 Spectrophotometer (Thermo Scientific, Wilmington, DE, USA). The extracted DNA was subjected to amplification using specific primers and sequencing. In PCR, the adjusted DNA concentration of approximately 10 ng was used for fungi and bacteria. For bacteria, standard Illumina sequencing primers (515 FB and 806 RB) were used to amplify the V3-V4 region of the 16S rRNA gene [31]. While ITS3mix and ITS4ngs primers were used to target the ITS2 region of fungi [32]. The amplicons were then subjected to electrophoresis on 0.8% agarose gel and subsequently sequenced using Illumina MiSeq technology 300 bp (MiSeq Reagent Kit v3, San Diego, CA, USA) with the sequencing service provided by Sangon Biotech (Shanghai, China) Co., Ltd. China. 

### 2.3. Metagenomic Sequence Data Analyses

After sequencing, a quality filter was applied to obtain high-quality reads (clean data) using the prinseq-lite tool, with a Phred quality cutoff value of 20 and a minimum length of 50 nucleotides for both strands. FLASH v1.2 [33] was used to combine forward and reverse reads, resulting in a single file concatenating paired and unpaired forward sequences. To identify operational taxonomic units (OTUs), sequences were clustered to a 97% identity using USEARCH [34] with the –usearch_global option. Low-quality sequences were eliminated using the –fastq filter command and the removal of chimeric sequences was performed using the –unoise2 command. The taxonomic classification for microbial OTUs was allocated by the SINA classifier using the SILVA database (http://www.arb-silva.de/ accessed on 15 July 2022) (bacterial 16S rRNA database) [35] and the RDP classifier using the UNITE database (https://unite.ut.ee/ accessed on 15 July 2022) (fungal ITS database) [36]. The multiple sequence alignment (MSA) of all OTUs was performed using MAFFT software (v7.490, https://mafft.cbrc.jp/alignment/software/ accessed on 16 July 2022) for further analysis. Furthermore, the abundance of OTUs was normalized using the samples with the least number of sequences as a standard value, and the subsequent normalized output was used for diversity analyses (alpha diversity and beta diversity). The alpha and beta diversity analysis (Shannon index) was performed using the QIIME software V1.7.0. Rarefaction and rank abundance curves were used to validate the depth and quality of the samples. Principal component analysis (PCA) was performed to identify the differences in bacterial and fungal community structures among tea leaves, soil, pupa intestine, and male and female moth intestine. Further, LEfSe analysis was carried out to identify the dominant biomarker with statistically significant abundance differences at the genus level in all samples using the LEfSe software (V1.0) (LDA score > 2) [37]. The Kyoto Encyclopedia of Genes and Genomes (KEGG) database (http://www.kegg.jp/kegg/pathway.html/ accessed on 18 July 2022) was employed to perform the annotation of differential metabolites and examine significantly enriched metabolic pathways [38]. 

### 2.4. Statistical Analysis

The Kruskal–Wallis test was used to examine the relative abundance of bacteria and fungi among different groups. Additionally, *t*-test and analysis of variance (ANOVA) were employed to evaluate the alpha diversity parameter (Shannon index) within each group and to identify any significant differences. 

## 3. Results

### 3.1. OTUs Annotation Results

**Figure 1:**
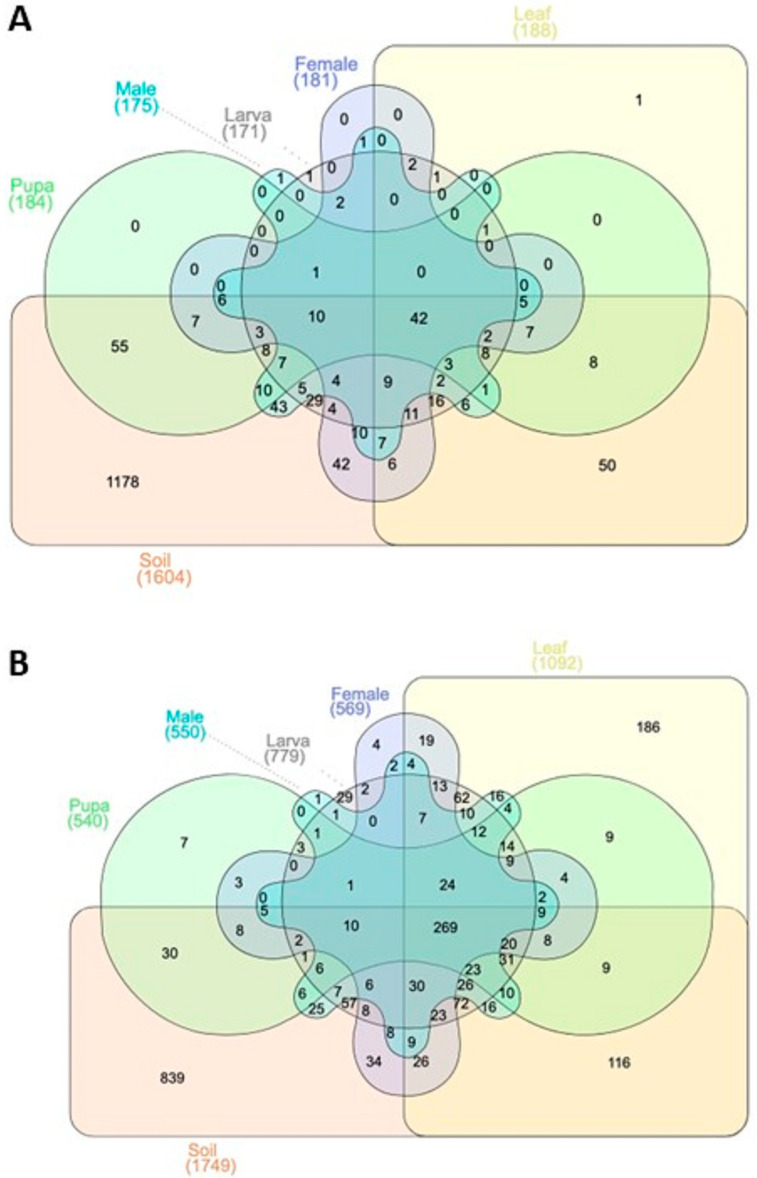
Venn diagrams illustrating the bacterial (**A**) and fungal (**B**) unique and common OTUs among all groups (soil, leaf, and tea moth larvae, pupae, female, and male gut).

**Figure 2:**
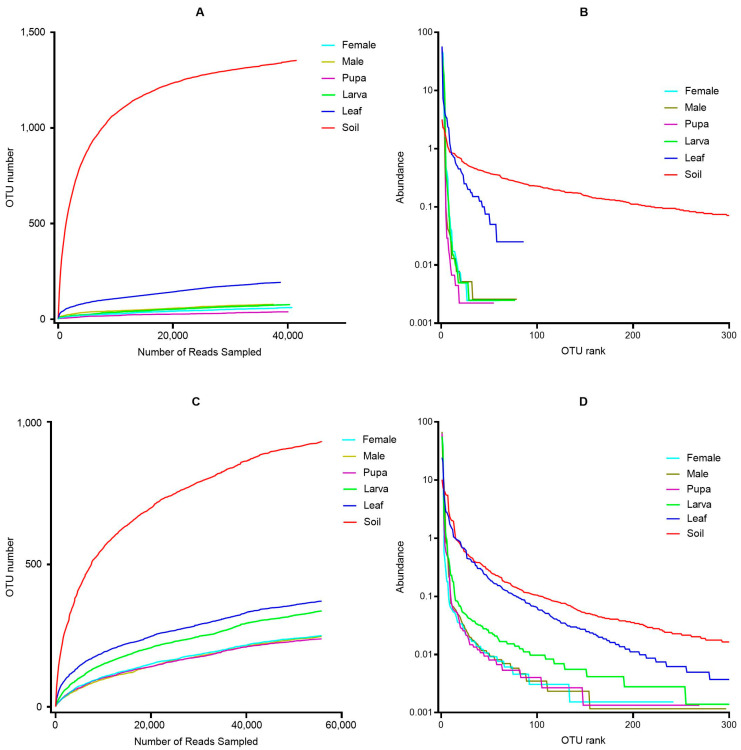
Rarefaction and rank abundance curves based on number of OTUs observed for bacteria (**A**,**B**) and fungi (**C**,**D**) among all treatment groups.

A Venn diagram was drawn to represent the shared and unique OTUs of bacterial and fungal communities among the studied groups, including soil, leaf, and tea moth gut samples (larvae, pupa, female, and male) (Figure 1A,B). Results showed that a large portion of unique OTUs (for bacterial and fungal communities) were found in rhizosphere soil samples. Moreover, the rarefaction and rank abundance curves showed the saturating number of OTUs, which were considered enough to identify the microbial diversity in all group samples (Figure 2A–D). For bacterial communities, a progressively flattened rarefaction curve showed that a great number of bacterial communities at a given depth were found in soil samples (Figure 2A). The rank abundance curve exhibited higher bacterial communities’ richness in soil samples than in leaf, larva, and male intestine group samples (Figure 2B). Rarefaction analysis for fungal communities revealed limited sequencing depth, potentially affecting diversity estimation and underrepresenting rare taxa in tea moth guts (Figure 2C). Future studies should consider high-throughput sequencing approaches to improve community resolution and capture broader fungal diversity. 

### 3.2. Alpha Diversity

**Figure 3:**
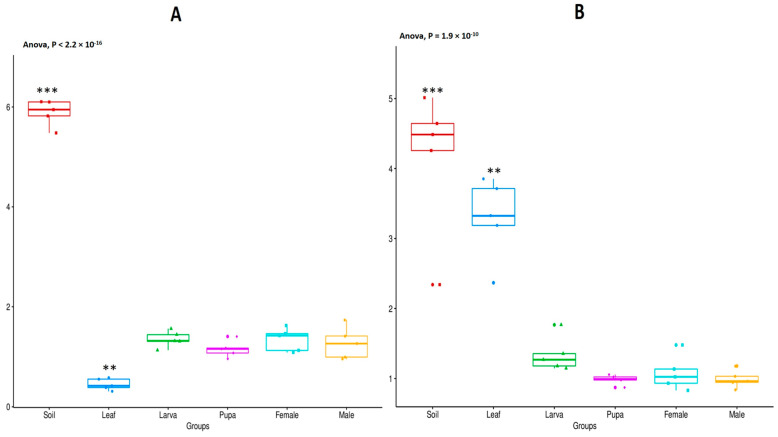
Alpha diversity (Shannon index) of (**A**) bacterial and (**B**) fungal communities among all groups (soil, leaf, and tea moth pupa, larvae, female, and male gut). A *t*-test was used to compare samples within each group, and the ANOVA test was used for comparisons between groups. In a boxplot, from bottom to top, there are lower bounds, lower quartiles (Q1), median values (Q2), upper quartiles (Q3), and upper bounds. Variables with ** and *** indicate that values differ significantly from other groups at *p* < 0.01 and *p* < 0.001, respectively.

The alpha diversity analysis (Shannon diversity index) was conducted to analyze the bacterial and fungal communities’ diversity in all group samples (soil, leaf, and tea moth larvae, pupae, female, and male gut) (Figure 3A,B). Results showed that the bacterial diversity was significantly higher (*p* < 0.05) in soil and lower (*p* < 0.05) in leaf group samples, while no significant differences (*p* > 0.05) were observed among the gut microbiomes of tea moth female, male, larva, and pupa stages (Figure 3A). Similarly, the Shannon diversity index also indicated that the fungal diversity was significantly higher (*p* < 0.05) in soil samples followed by the leaf samples (Figure 3B). Meanwhile, there were no differences (*p* > 0.05) in the fungal diversity of tea moth gut in male, female, larva, and pupa stages. Overall, soil and leaf sample diversity significantly (*p* < 0.05) varied compared to tea moth gut samples.

### 3.3. Relative Abundance of Bacterial and Fungal Communities

**Figure 4:**
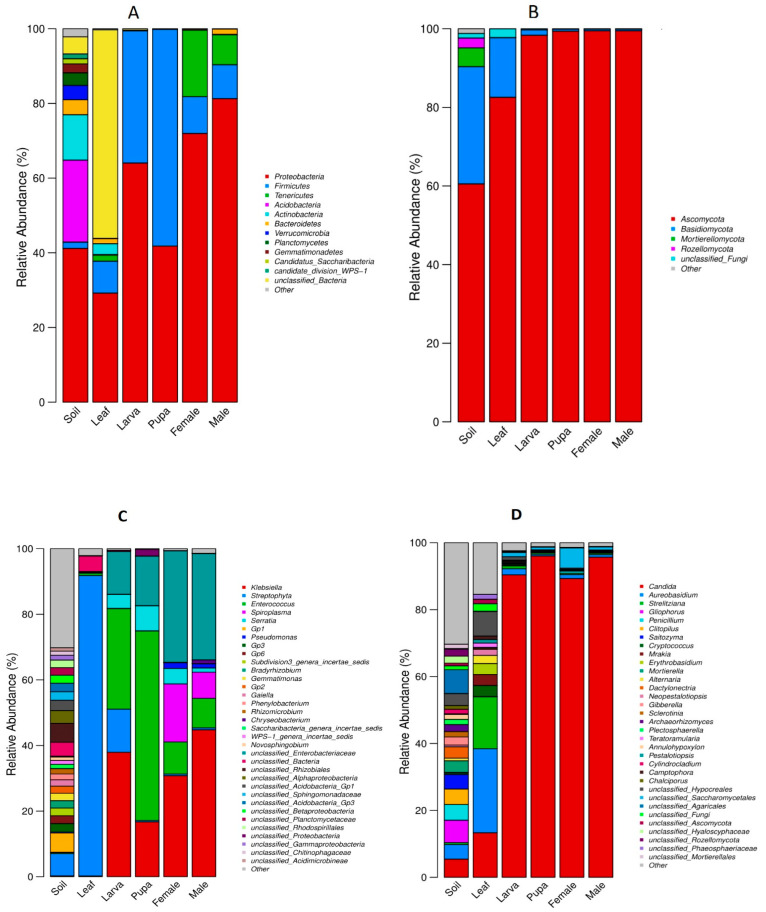
Relative abundance of (**A**) bacteria and (**B**) fungi at the phylum and (**C**) bacteria and (**D**) fungi at genus level among all groups (soil, leaf, tea moth larvae, pupa, female, and male gut).

A relative abundance of top bacterial and fungal community members of soil, leaf, and tea moth larvae, pupae, female, and male gut was observed and represented in the form of a histogram (Figure 4A–D). The top three bacterial phyla were Proteobacteria, Firmicutes, and Tenericutes, but their distribution varied among all group samples (Figure 4A). Among these, Proteobacteria was highly abundant in all group samples followed by Firmicutes, which was abundant in different developmental stages of the moth (tea moth larva, pupa, female, and male gut). Further, the relative abundance of Tenericutes was high in female and male gut, whereas tea moth larva, pupa gut, leaf, and soil samples had negligible amounts of this phylum. Similarly, the top three fungal phyla were Ascomycota, Basidiomycota, and Mortierellomycota (Figure 4B). Among these top three phyla, the relative abundance of Ascomycota was higher in all group samples, followed by Basidiomycota, which was abundant in soil, leaf, and larva gut samples. Further, the relative abundance of Basidiomycota in the tea moth gut was comparatively higher in larval gut than in pupa, female, and male gut. However, the phylum Mortierellomycota was found only in soil group. Moreover, the Kruskal–Wallis (KW) test revealed significant differences (*p* < 0.05) among the top abundant bacterial and fungal phyla in all group samples (soil, leaf, and tea moth larvae, pupae, female, and male gut) (Supplementary Figures S1A–K and S2A–K). Further, the top three bacterial genera were *Klebsiella*, *Streptophyta*, and *Enterococcus*, with distinctive relative abundance among all group samples (Figure 4C). The results showed a higher relative abundance of *Klebsiella* in the male gut as compared to larval, pupa, and female gut samples. However, *Streptococcus* was highly abundant in leaf samples, but its relative abundance was lower in other group samples (soil, tea moth larva, pupa, female, and male gut). Further, *Enterococcus* had a higher relative abundance in the pupa gut compared to other tea moth developmental stages (larva, female, and male gut), while soil and leaf group samples had a negligible amount of this genus. Meanwhile, the top three fungal genera were *Candida*, *Aureobasidium*, and *Strelitziana* (Figure 4D). Among them, *Candida* exhibited a high relative abundance in all group samples (soil, tea moth larva, pupa, female, and male gut). Further, *Candida* abundance was comparatively higher in pupa and male gut, followed by the larva, female, leaf, and soil samples. However, *Aureobasidium* and *Strelitziana* were dominant in leaf samples but showed no significant differences in among group samples (soil, tea moth larva, pupa, female, and male gut). Further, the Kruskal–Wallis (KW) test revealed significant differences (*p* < 0.05) among the top abundant bacterial and fungal genera in all group samples (soil, leaf, and tea moth larvae, pupae, female, and male gut) (Supplementary Figures S3A–K and S4A–K). 

### 3.4. Beta Diversity

**Figure 5:**
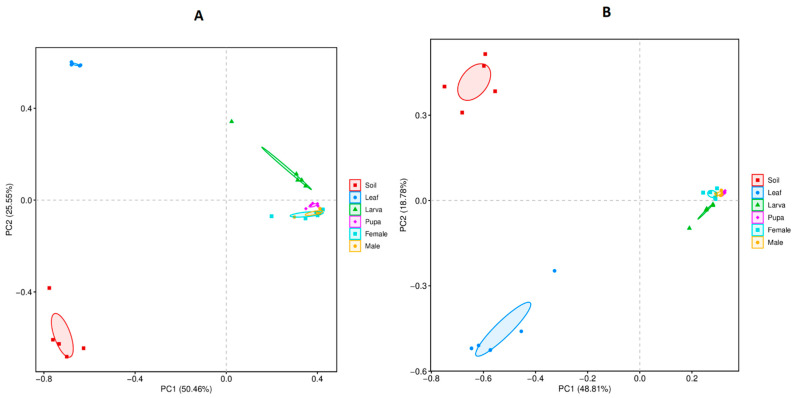
PCA analysis based on (**A**) bacterial and (**B**) fungal communities showing the differences and similarities among all group samples (soil, leaf, and tea moth larvae, pupa, female, and male gut).

Principle component analysis (PCA) showed similarities and differences in the bacterial and fungal community between all group samples (soil, leaf, tea moth larvae, pupae, female, and male gut) (Figure 5A,B). For bacterial communities, PC1 and PC2 accounted for 50.46% and 25.55% of total variation, respectively (Figure 5A). The bacterial communities were more distinctive in soil and leaf group samples than the different developmental stages of the moth (larva, pupa, female, and male gut) as they were segregated. Among the different developmental stages of the tea moth, larval gut bacterial communities were distinctive as they were not clustered with other tea moth group samples. For fungal communities, PC1 and PC2 explained a total of 48.81% and 18.78% variation, respectively (Figure 5B). The soil and leaf group samples showed a highly diverse fungal community, while tea moth larva, pupa, female, and male gut exhibited more similarities as these group samples were closely clustered. Within different developmental stages of the tea moth, the larval gut presented more distinctive fungal communities compared with the pupa, female, and male gut. Overall, bacterial and fungal community differences were evident between soil and leaf based on their separate clusters than within different developmental stages of tea moth (larva, pupa, female, and male gut). 

### 3.5. Biomarker Taxa of Bacterial and Fungal Communities

**Figure 6:**
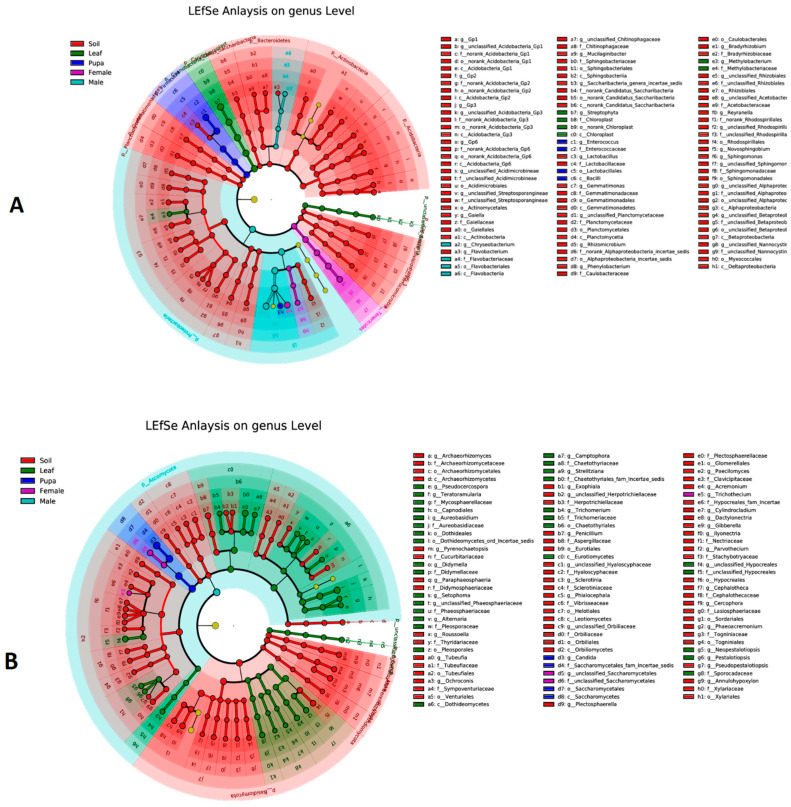
LEfSe analysis: (**A**) bacterial and (**B**) fungal communities showing biomarker taxa among all groups (soil, leaf, and larvae, pupa, female, and male gut).

LEfSe analysis was performed to predict the biomarker taxa of bacterial and fungal communities among all group samples (soil, leaf, and tea moth larvae, pupae, female, and male gut) at the genus level (Figure 6A,B). For bacterial communities, soil samples had the highest number of biomarker taxa at the genus level, including *Acidobacteria*, *Gaiella*, *Flavobacterium*, *Mucilaginibacter*, and *lactobacillus*, etc., mainly following the leaf samples (*Streptophyta* and *Methylobacterium*), pupa (*Enterococcus*), and male (*Chryseobacterium*) (Figure 6A). For fungal communities, mainly *Archaeorhizomyces*, *pyrenochaetopsis*, *Exophiala*, and *Phialocephala*, etc., were detected as dominant biomarker genera, following the leaf samples (mainly *Pseudocercospora*, *Teratoramularia*, *Didymella*, and *Setophoma*, etc.), pupa (*Candida*), and female (unclassified_*Saccharomycetales*) (Figure 6B). The results showed that dominant biomarkers showing differences between bacterial and fungal communities were more distinct in rhizosphere soil and leaf samples. 

### 3.6. Functional Enrichment Analysis

**Figure 7:**
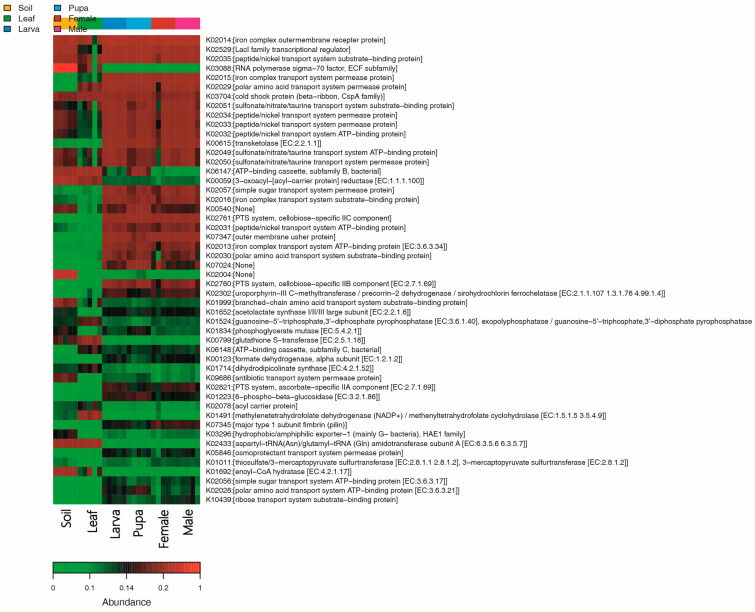
The relative abundance of functionally enriched pathways bacteria among all groups (soil, leaf, and tea moth larvae, pupa, female, and male gut).

KEGG pathway analysis was performed to identify the significantly enriched metabolic pathways among all group samples (soil, leaf, and tea moth larvae, pupa, female, and male gut) (Figure 7). For the bacterial communities, the most enriched metabolic pathways were iron complex outer-membrane receptor protein, Lacl family transcriptional regulator, peptide/nickel transport system substrate-binding protein, and RNA polymerase sigma-70 factor, ECF subfamily in soil samples. ATP-binding cassette, subfamily B, and bacterial serine/threonine protein kinase were most abundant in leaf samples. Meanwhile, iron complex outer-membrane receptor protein, PTS system, and cellobiose-specific IIC component were functionally enriched in tea moth larva, pupa, female, and male gut samples. 

## 4. Discussion

Insects are the major cause of the world’s annual agricultural yield losses, accounting for 40% of yield losses, resulting in monetary losses of approximately USD 220 billion (https://www.fao.org, accessed on 5 December 2022). Insects have four distinct developmental stages: egg, larva, pupa, and adult, which infest the plants primarily for food, sustenance, and shelter for reproduction [39]. Tea grain moth is a common insect pest of tea gardens in China. It mainly defoliates plant leaves, sucks nutrients, destroys leaf mesophyll, and feeds on the bark and twigs of tea plants [19]. Tea garden moths are the most destructive defoliators that feed on young leaves and infest thousands of hectares of tea gardens, negatively impacting tea production [40]. The insect’s microbiome plays an important role in mediating its health and strength. Studies have shown that leaf-eating insects can also acquire some microbes from plant leaves and soils and integrate them into their gut microbiome [23]. Moreover, it has been reported that bacteria between leaves and gut significantly varied among caterpillar species, suggesting that some species have transient bacterial microbiota. These findings suggest the complexity of the factors shaping the gut microbiota, while highlighting interspecific differences in microbial colonization within the same insect species [41,42]. Therefore, studying the microbiome of moths is of paramount importance for understanding the dynamics of the microbial community in tea leaves, rhizosphere soil, and the different developmental stages of moths. Recently, the advancement of next-generation sequencing techniques has led to an increasing number of studies to assess the microbial diversity of insects [43,44,45,46]. 

In the present study, the diversity of bacterial and fungal communities in tea leaves, rhizospheric soil, and tea moth larvae, pupae, female, and male guts was investigated using 16S rRNA and ITS gene sequencing. Our findings revealed the intricate dynamics of the microbial communities in all samples. In contrast to soil microbiome, which had more distinct OTUs, moth gut and tea leaves had more common microbial communities, indicating that a major portion of the gut microbiome was acquired from leaves. Considerable changes in the bacterial and fungal communities between the soil, leaf, and tea moth gut samples were detected based on the Shannon index. However, gut microbiomes of tea moth larvae, pupae, females, and males showed no significant variations. These findings are consistent with previous studies that also reported significantly higher microbial diversity in soil than in leaf and insect gut samples [47]. However, during developmental stages, the microbial diversity was also significantly higher in larvae than in pupae and adults [47]. A previous study revealed that the gut microbiota of Lepidoptera is primarily leaf-derived, and low in abundance, suggesting their transient nature [41]. Furthermore, beta diversity analysis of bacterial and fungal communities revealed that soil and leaf group samples showed greater differences as compared to the guts of moth larvae, pupae, females, and males. These findings are in agreement with earlier studies that also observed variations between the insect developmental phases and the microbial richness of plant leaves [23]. Additionally, *Agriophara rhombata’s* larval gut had greater variations in bacterial and fungal communities during development than pupa, female, and male guts in terms of microbial diversity, as also reported earlier [23,44]. This higher diversity is mainly attributed to the fact that the larva actively feeds on microbe-rich leaves compared to adults, which rely more on liquid diets [48].

LEfSe analysis was performed to predict the biomarker taxa of bacterial and fungal communities, and the results showed that dominant biomarkers explaining differences between bacterial and fungal communities were more prevalent in soil and leaf samples, which could be attributed to the higher and more diverse microbial communities of soil and leaf samples than tea moth gut samples. For bacterial biomarkers, the genus *Acidobacteria* was documented as the most dominant biomarker in soil samples. In previous studies, *Acidobacteria* that were abundant in soil were also found in the larval gut and contributed to the degradation of plant polymers (cellulose and xylan) for nutrient acquisition [49]. In leaf samples, *Streptophyta* and *Methylobacterium* were detected as predominant genera. Previously, Streptophyta and Methylobacterium had also been detected in tobacco leaf samples treated with biological control agent (BCA) against tobacco blight, and Streptophyta was increased significantly, indicating its potential role in inhibiting the disease [50]. *Enterococcus* and *Chryseobacterium* were found as biomarker genera in tea moth males and pupae. Enterococcus was predominantly found in the gut microbiome of the leafworm *Spodoptera littoralisa* [48,51]. Enterococcus and *Chryseobacterium* have also been detected in the midgut microbiome of the potato tuber moth [52]. For fungal communities, genera such as *Archaeorhizomyces* and *Exophiala* were dominant taxa in soil samples. Previous studies have shown that Archaeorhizomycetes is a prominent component of the soil rhizosphere, accounting for up to one-third of the total fungal population [53], and *Exophiala* has the potential as a biomarker species indicative of restoration progress in polluted soils [54]. Leaf samples contained the genus *Pseudocercospora*, which has been documented as a foliar pathogen in previous metagenomic studies [55,56]. The genus *Didymella* was also detected in leaf samples as biomarker taxa. *Didymella* spp. have been shown to cause leaf spots in tea plants, resulting in great economic losses in China [57]. 

KEGG pathway analysis identified the most enriched metabolic pathways, including iron complex outer-membrane receptor protein, Lacl family transcriptional regulator, peptide/nickel transport system substrate-binding protein, and RNA polymerase sigma-70 factor, ECF subfamily in soil samples. These functionally enriched pathways have also been detected in soil, as reported previously [58]. ATP-binding cassette, subfamily B, and bacterial and serine/threonine protein kinase were functionally enriched pathways in leaf samples. These genes are abundant in plants and play a key role in plant defense by mediating signal transduction [59], whereas iron complex outer-membrane receptor protein, PTS system, and cellobiose-specific llC component were functionally enriched in larval, pupal, female, and male intestines. Iron complex outer-membrane receptor protein is important for signaling and cellular processes during the different developmental stages of insects [60]. The PTS system helps bacteria to maintain different sugar levels and is involved in carbon metabolism. The high abundance of cellobiose-specific IIC components in the insect gut is involved in the metabolism of cellobiose derived from plants [61]. Overall, it can be suggested that the microbial differences were higher among soil, leaves, and moth developmental stages; however, no significant differences were observed within developmental stages (larva, pupa, female, and male gut). 

## 5. Conclusions

The present study concluded that the bacterial and fungal communities of soil and leaf of *Camellia sinensis* (tea plant) had significantly higher diversity and functional enrichment of metabolic pathways than the gut microbiome of *Agriophara rhombata* (tea moth) at its different developmental stages (larvae, pupae, female, and male). These findings contribute to our understanding of the gut microbiota of the tea moth and its potential application in the development of pest control strategies.

## Data Availability

The sequenced data was submitted to the NCBI under accession SRA, Project no. PRJNA983257.

## Supplementary Materials

The following supporting information can be downloaded at: https://www.mdpi.com/article/10.3390/microorganisms11092188/s1: Table S1, Completely randomized design (CRD) showing a total of six treatments, each with five replicates; Figure S1A–K, Kruskal boxplot showing the significant differences in top abundant bacterial phyla; Figure S2A–K, Kruskal boxplot showing the significant differences in top abundant fungal phyla; Figure S3A–K, Kruskal boxplot showing the significant differences in top abundant bacterial genera; Figure S4A–K, Kruskal boxplot showing the significant differences in top abundant fungal genera.
