# Supplementary material for: Diversity and Abundance of Bacterial and Fungal Communities Inhabiting Camellia sinensis Leaf, Rhizospheric Soil, and Gut of Agriophara rhombata
Source: Microorganisms. 2023 Aug 30;11(9):2188. doi: 10.3390/microorganisms11092188 (PMC10536862; doi:10.3390/microorganisms11092188)
Supplement: Supplementary file 1 [file microorganisms-11-02188-s001.zip › microorganisms-2494183-supplementary.pdf]

Supplementary material

Supplementary Table S1. Completely randomized design (CRD) Showing a total of six treatments, each with five replicates.

No.	Treatments	Replicates
1	SOIL (YK10-T)	YK10-T-1, YK10-T-2，YK10-T-5，YK10-T-7，YK10-T-8
2	TEA LEAF (YK10-Y)	YK10-Y-3, YK10-Y-5，YK10-Y-7，YK10-Y-9，YK10-Y-10
3	Larval intestine (YK10-3)	YK10-3-1, YK10-3-2，YK10-3-3，YK10-3-4，YK10-3-5
4	Pupal intestinal tract (YK10-5)	YK10-5-1, YK10-5-2，YK10-5-3，YK10-5-4，YK10-5-5
5	Female moth intestine (YK10-C), (1-5)	YK10-C-1, YK10-C-2，YK10-C-3，YK10-C-4，YK10-C-5
6	Male moth intestine (YK10-C), (6-10)	YK10-C-6, YK10-C-7，YK10-C-8，YK10-C-9，YK10-C-10

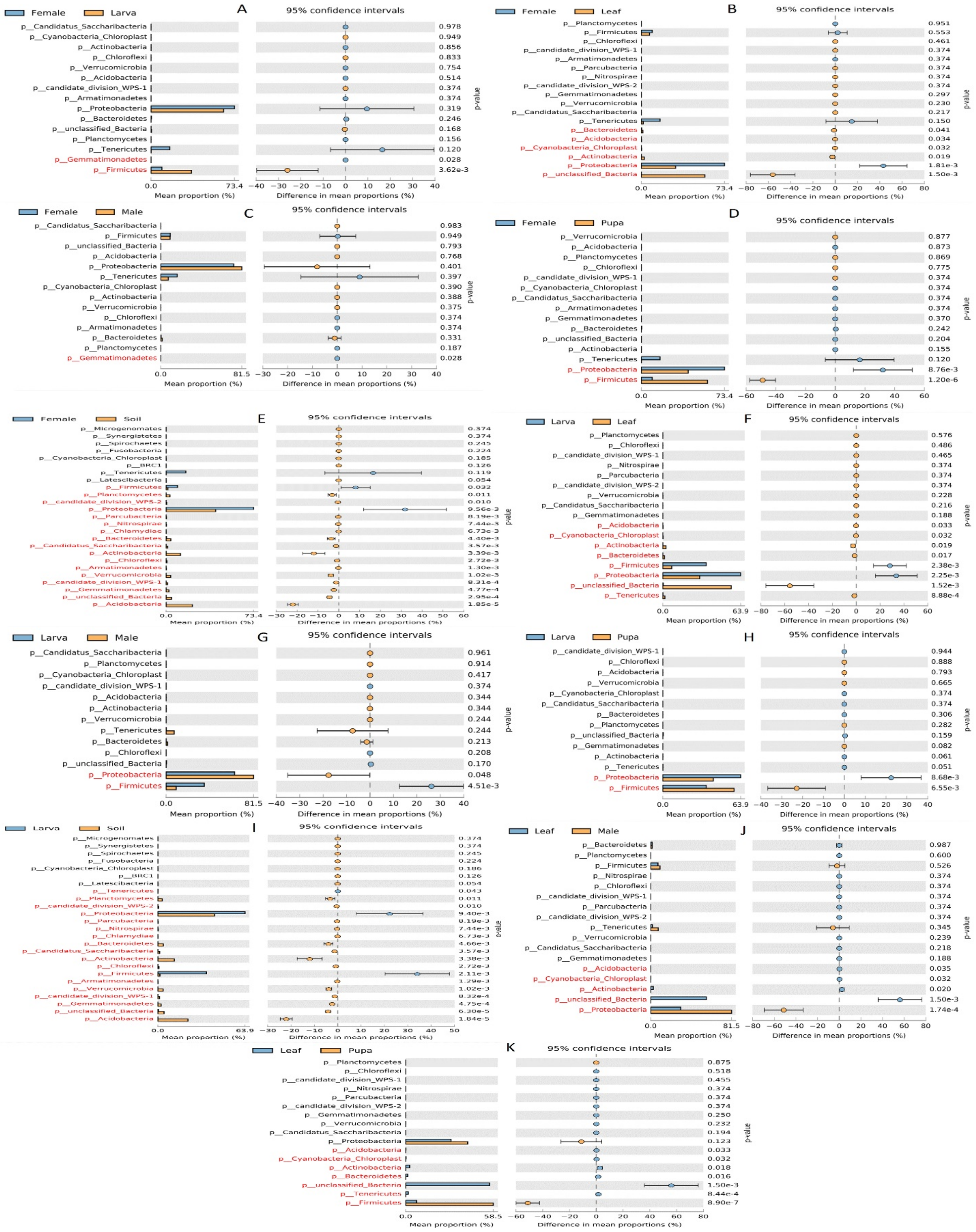

Figure S1. (**A–K**) Kruskal boxplot showing the significant differences in top abundant bacterial phyla

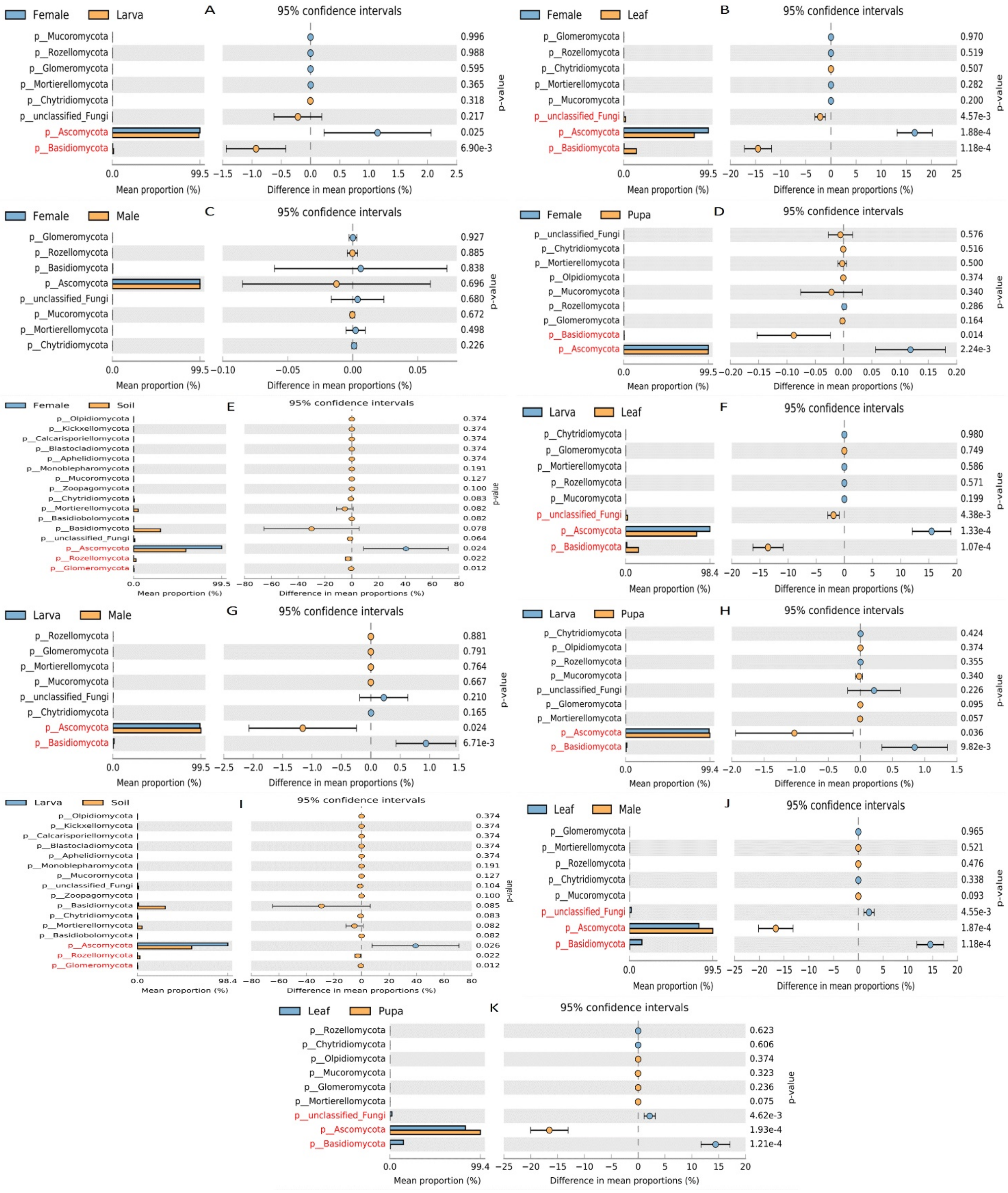

Figure S2. (**A–K**) Kruskal boxplot showing the significant differences in top abundant fungal phyla

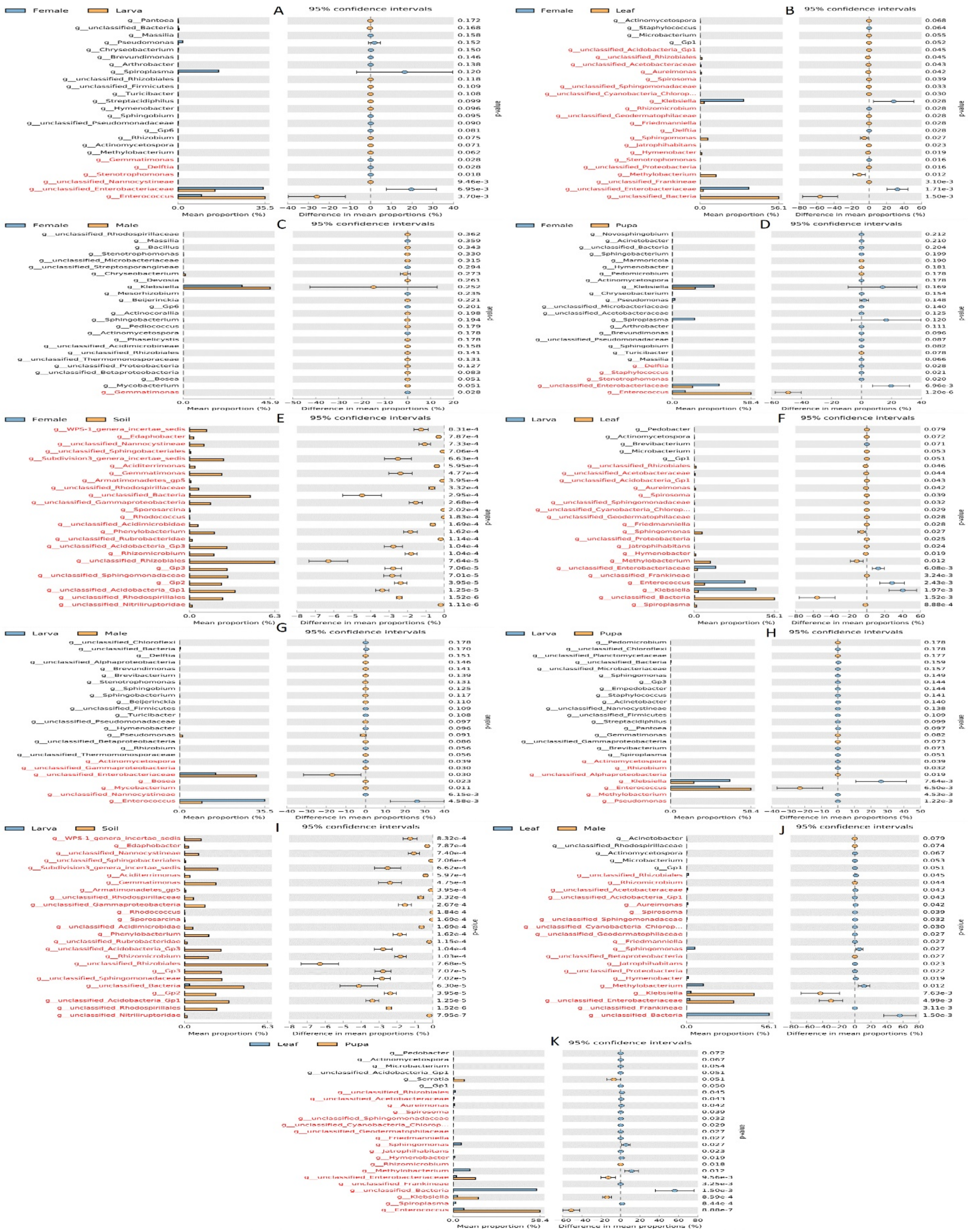

Figure S3. (**A–K**) Kruskal boxplot showing the significant differences in top abundant bacterial genera

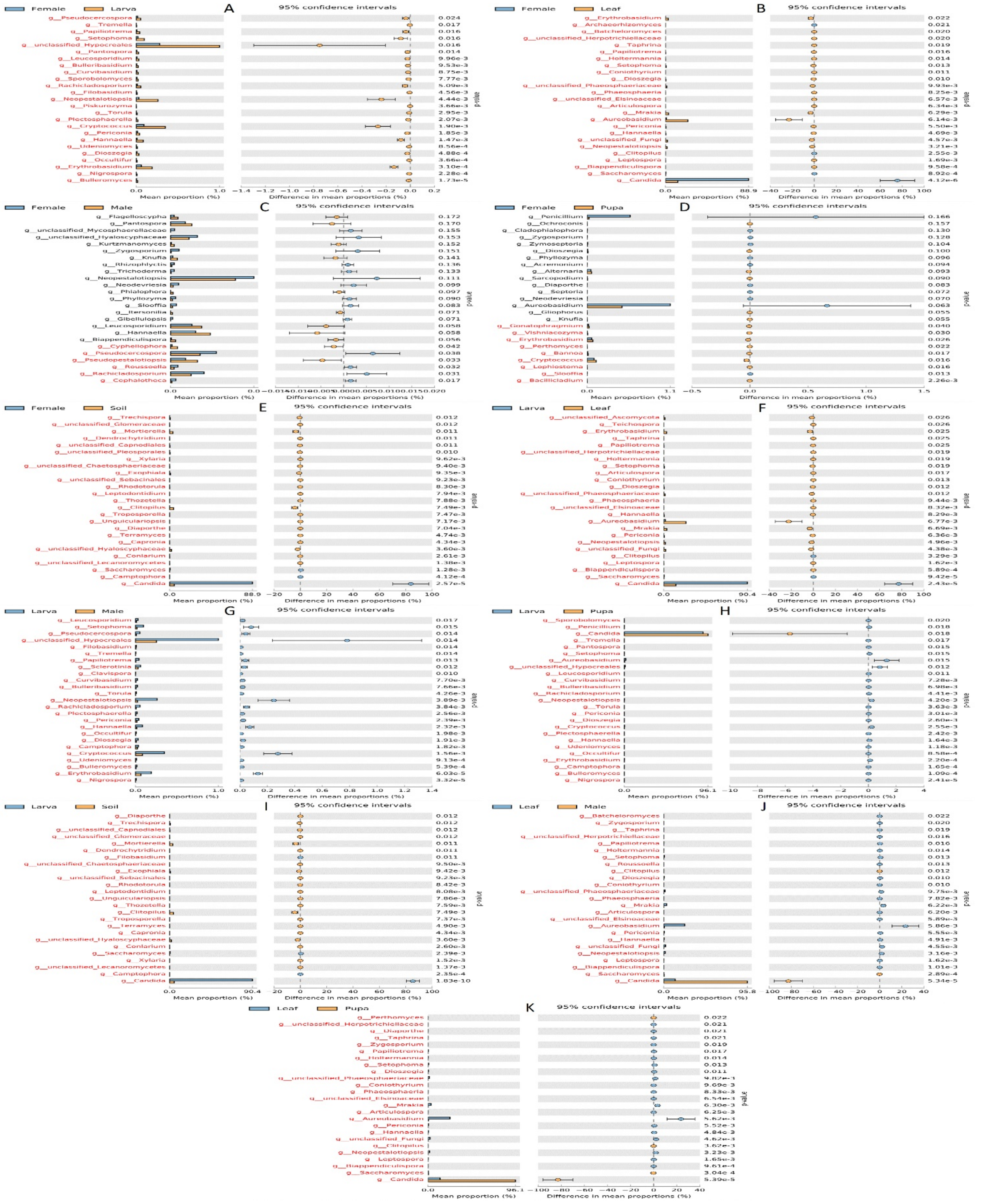

Figure S4. (**A–K**) Kruskal boxplot showing the significant differences in top abundant fungal genera
